# Focal acute cholecystitis misdiagnosed as gallbladder carcinoma

**DOI:** 10.1007/s40477-025-00995-z

**Published:** 2025-02-13

**Authors:** G. L. D’Alessandro, G. Ancona, C. Dellafiore, A. Raimondi, D. Gangemi, G. Arpa, G. Poma, E. Above

**Affiliations:** 1 Department of Internal Medicine and Therapeutics, University of Pavia, Pavia, Italy; 2 Diabetes Research Institute, IRCCS San Raffaele Hospital, Milan, Italy; 3 Ultrasound Unit, Infectious Diseases Department, San Matteo Hospital Foundation, Pavia, Italy; 4 Unit of Diabetology, Ospedale Di Circolo E Fondazione Macchi, Varese, Italy; 5 Unit of Anatomic Pathology, ICS Maugeri-IRCCS SpA SB, Pavia, Italy

**Keywords:** Acute cholecystitis, Gallbladder carcinoma, Gallbladder wall-thickening, Contrast-Enhanced Ultrasound in Non-Hepatic Applications, Biliary tract disease

## Abstract

Thickening of the gallbladder wall is often associated with acute or chronic cholecystitis, adenomyomatosis and gallbladder carcinoma or seen in the context of liver and systemic diseases (acute hepatitis, cirrhosis, sepsis). Here we present a case of a 61 y.o. man with focal thickening of the gallbladder wall, in whom all imaging techniques were inconclusive. Pathological examination of the resected gallbladder revealed acute-on-chronic cholecystitis. We describe focal acute cholecystitis in absence of the classic clinical and imaging findings (Murphy’s sign, fever, gallstones, hydrops, pericholecystic fluid) and mimicking a gallbladder carcinoma.

## Introduction

A thick wall gallbladder is a common but unspecific finding, due to a wide spectrum of different pathologic conditions, involving primarily or secondarily the gallbladder. One of the most common classifications distinguishes between diffuse and focal thickening [1]. The two most important causes of diffuse gallbladder wall thickening are acute and chronic cholecystitis. Focal thickening of the gallbladder wall has a narrow differential diagnosis, including neoplastic and non-neoplastic processes [1]. Primary gallbladder carcinoma is a rare but fearsome etiology of gallbladder wall pathology and manifests either as a diffuse lesion replacing the gallbladder lumen and infiltrating the adjacent parenchyma, or as a focal lesion (polypoid mass or wall thickening). The distinction between benign and malignant lesions of the gallbladder wall is of outmost importance, but some degree of diagnostic overlap exists between them [2]. We present a case of a 61 y.o. male, with history of urothelial carcinoma, who presented with a marked focal thickening of the gallbladder wall at imaging studies.

## Case presentation

**Fig. 1:**
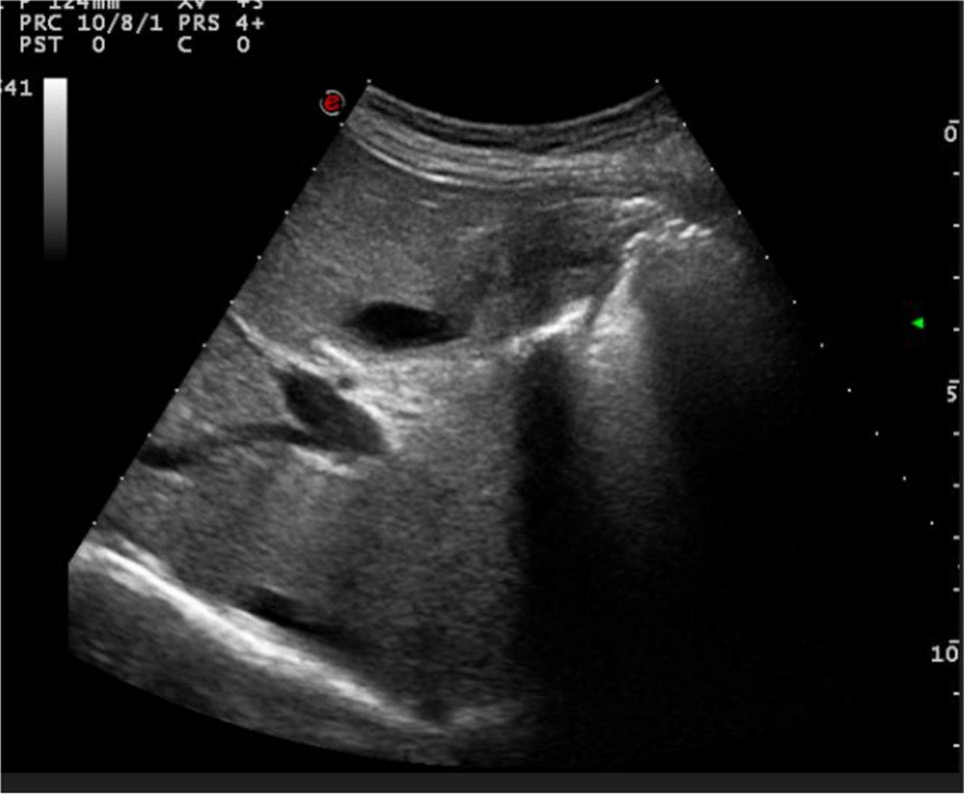
B-mode ultrasound of the gallbladder showing pathologic wall thickening (14 mm) localized in the body and fundus of the gallbladder, with no evidence of gallstones

**Fig. 2:**
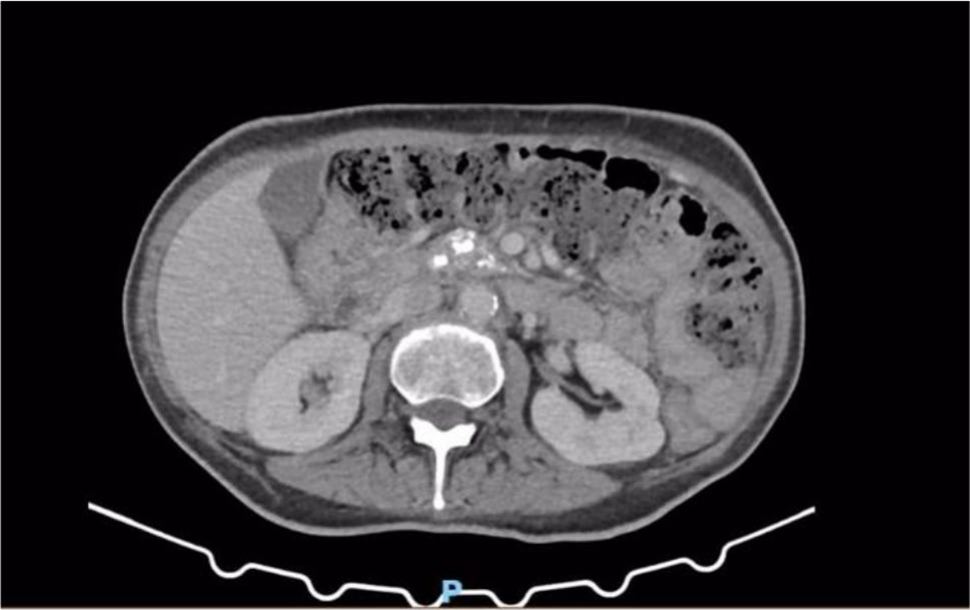
Abdominal CT scan before onset of symptoms showing no alterations of the gallbladder wall

**Fig. 3:**
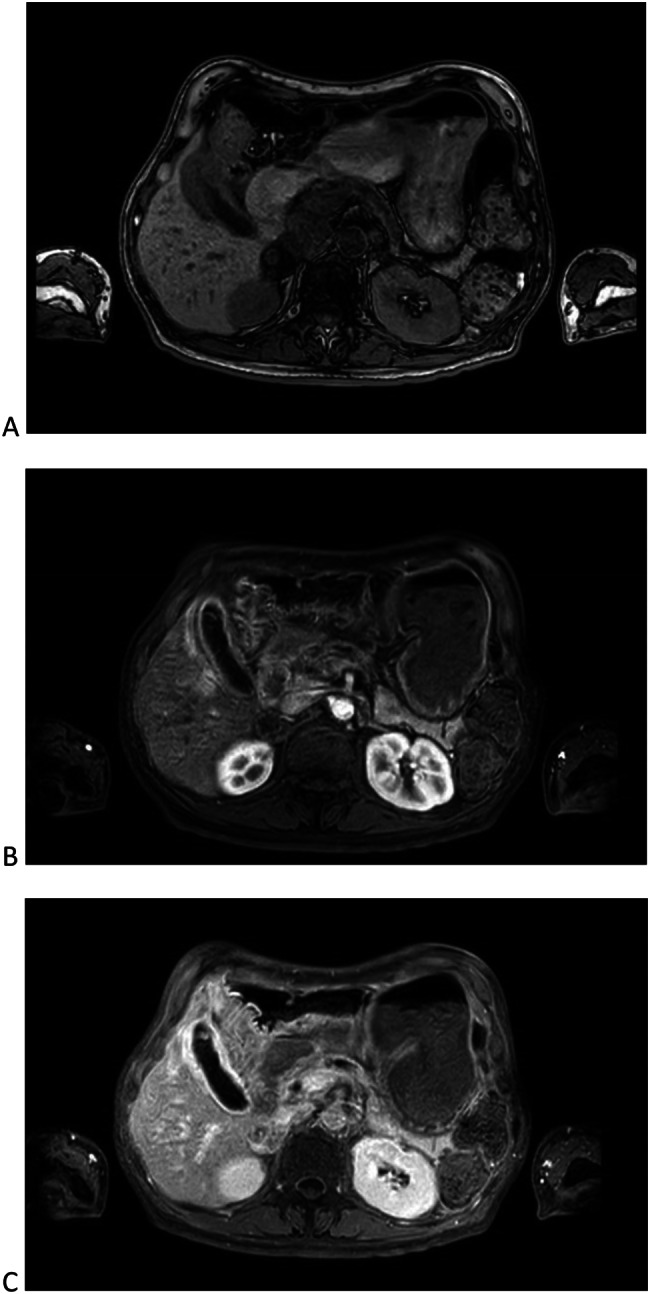
Abdomen MRI findings: **A** T1-weighted sequence showing irregular thickening of the distal body and fundus of the gallbladder wall (maximum diameter 18 mm). The thickness was T1 hypo-intense to the surrounding liver. **B** Early arterial enhancement of the wall thickening after contrast agent injection, with reactive hyperemia of the adjacent liver parenchyma. C) Venous phase documents retention of the contrast agent in venous phase

**Fig. 4:**
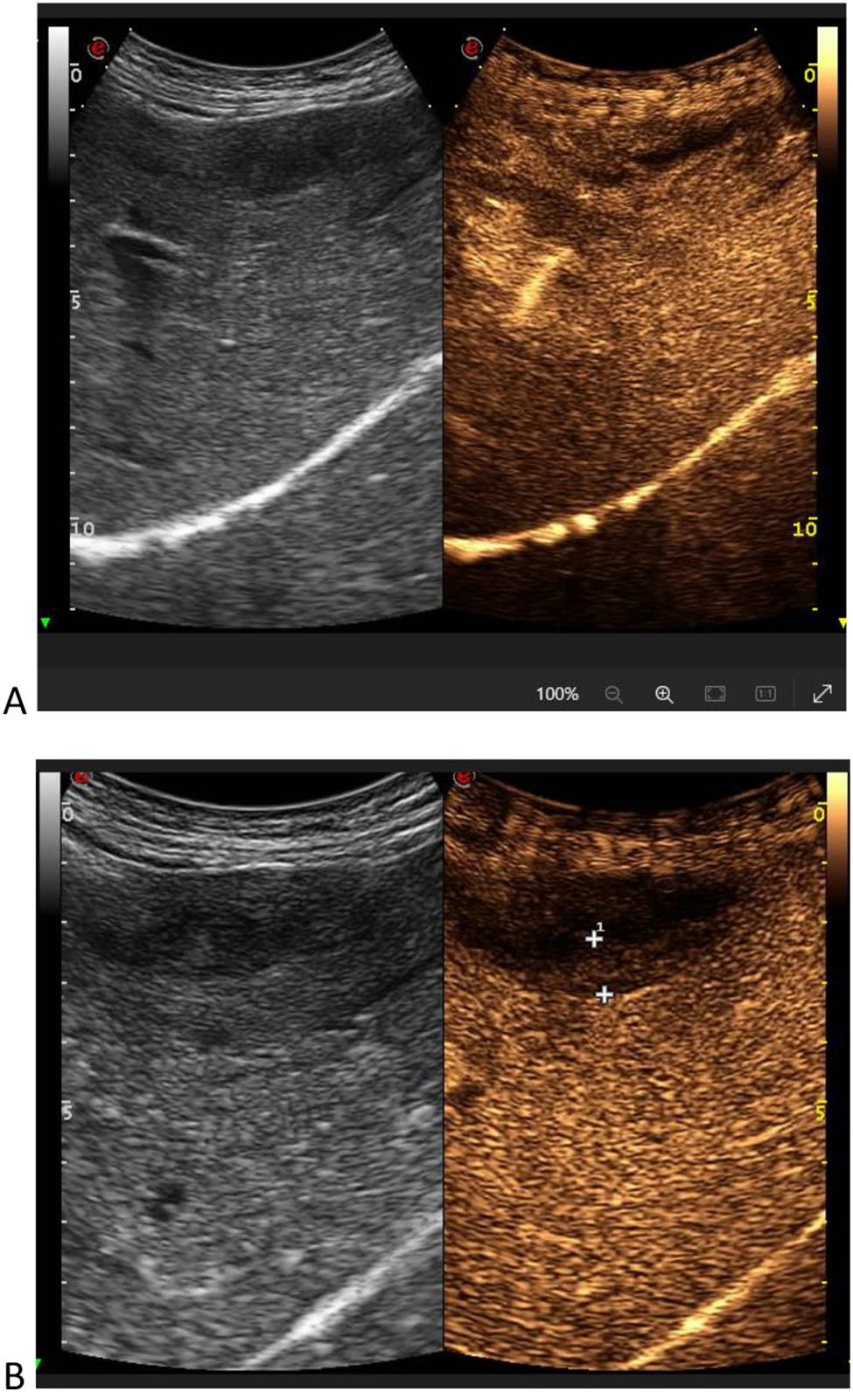
CEUS showing hyper-enhancement of the gallbladder wall in arterial phase (**A**), followed by wash-out in late venous phase (**B**)

A 61 y.o. Italian male sought medical attention in May 2021 because of nausea and vomiting. The patient did not report fever or abdominal pain. At the time of the visit, vital signs were normal and physical examination of the abdomen was negative. He was a heavy smoker and his past medical history included: urothelial bladder carcinoma treated with chemotherapy and surgery (cystectomy, pelvic lypmhoadenectomy and ureteroileocutaneostomy) three years before, type 2 diabetes with microvascular complications, chronic pancreatitis, hypertensive heart disease, liver steatosis, multifactorial anemia, right adrenal adenoma, osteoporosis with multiple vertebral collapses. The patient was taking the following drugs: insulin (basal-bolus), antiplatelet, ACE inhibitor, pancreatic enzymes, vitamin D. The blood tests showed transaminitis (GOT 225 U/L, GPT 393 U/L) and increased ALP (384 U/L) and GGT (135 U/L), normal total and differential WBC (5920/mm3) and preserved renal function (serum creatinine 0.63 mg/dl). The B-mode abdominal ultrasound showed a pathologic thickening (14 mm) of the gallbladder wall localized in the body and fundus of the gallbladder, with no evidence of gallstones (Fig. 1). A routine CT scan of the abdomen performed two months before, scheduled for oncologic follow-up, did not report any alteration of the gallbladder (Fig. 2). For a better characterization of the lesion, a contrast-enhanced MRI of the abdomen was performed, showing an irregular thickening of the distal body and fundus of the gallbladder wall (maximum diameter 18 mm), with nodular aspects. The thickened wall was T1 hypo-intense to the surrounding liver. Early enhancement after contrast agent injection was noted, affecting also the gallbladder bed (as in case of liver involvement), with retention of the contrast and hyperenhancement in tardive phases (Fig. 3A, B, C). No signs of adenomyomatosis, xanthogranulomatous cholecystitis, gallstones or disruption of the mucosal line were present and liver parenchyma did not show focal lesions. The MRI findings raised the suspicion of gallbladder malignancy. CEUS was also performed, confirming the presence of the wall thickening, which presented early enhancement in arterial phase, followed by a wash-out during venous phase (Fig. 4A, B), supporting the MRI findings. Tumour markers (CEA, CA19.9, AFP) were negative. After 1 month since the onset of the symptoms, the patient started feeling better, with no other episodes of nausea and vomiting. Blood tests were repeated, with normalization of liver and cholestasis enzymes. After surgical evaluation, the patient was scheduled for laparoscopic cholecystectomy, performed in July 2021.

**Fig. 5:**
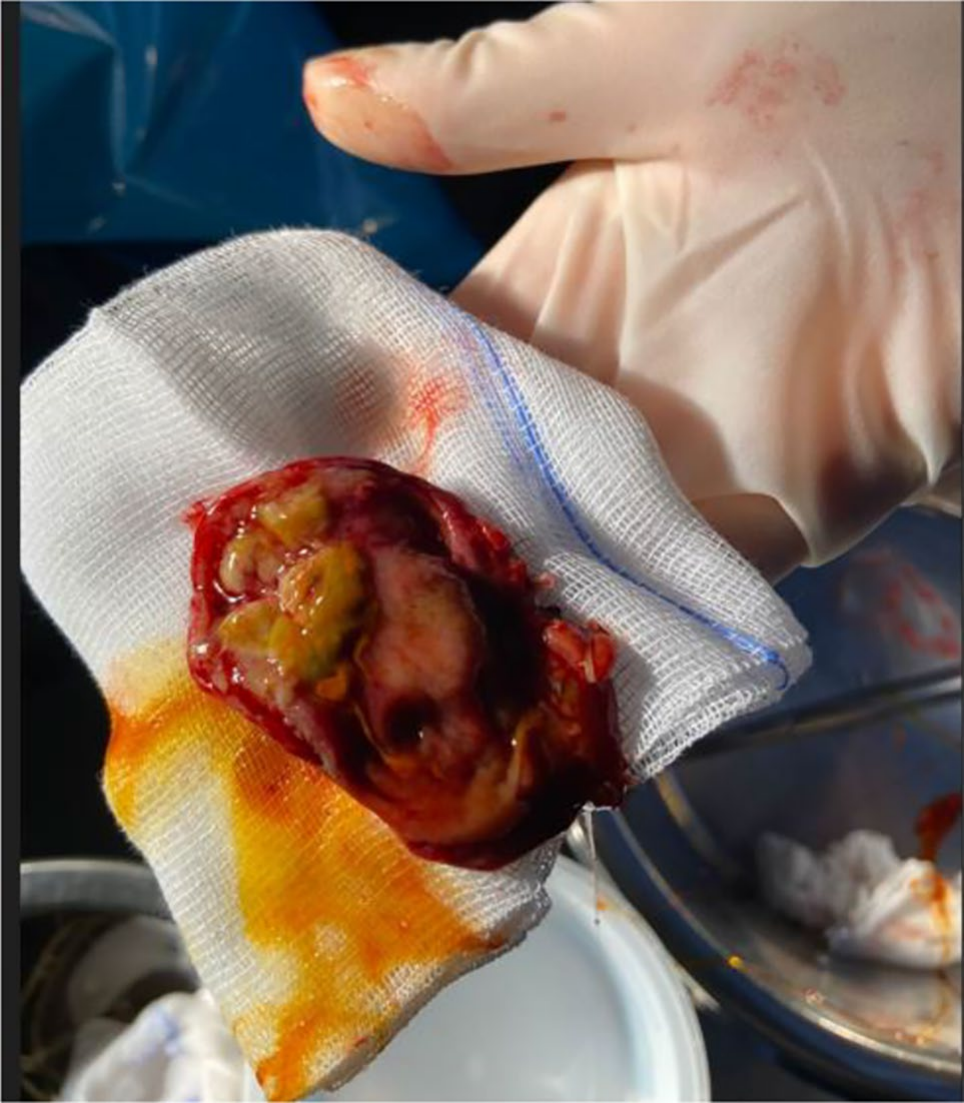
Surgical specimen with the focal wall thickening with characteristic nodular aspect of the intraluminal surface

**Fig. 6:**
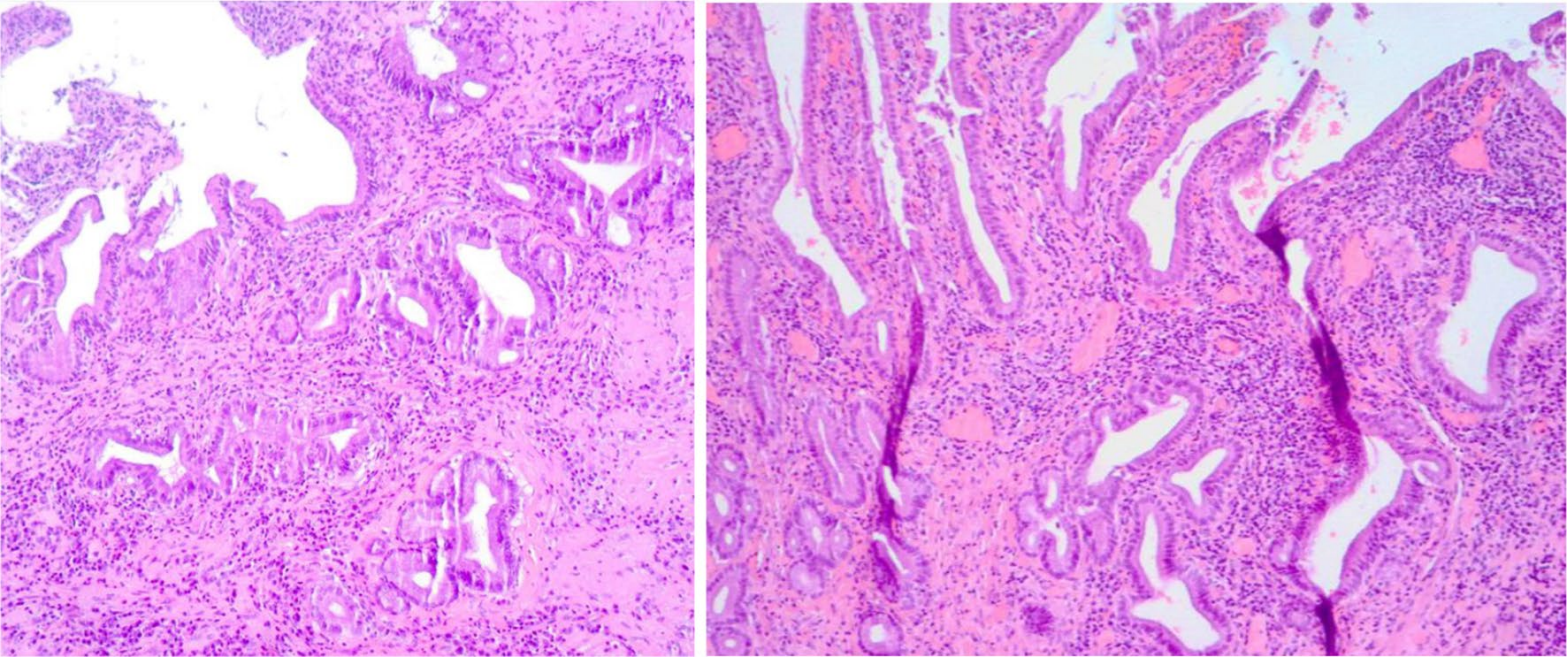
Pathologic examination showing severe inflammatory infiltrate composed by lymphocytes, neutrophils, plasma cells and eosinophils. Focal ulceration and necrosis and focal adenomyosis were noted, without presence of atypic cells of malignant significance

The intraoperative ultrasound examination confirmed the focal thickening of the gallbladder wall, without infiltration of the liver and absence of regional lymphoadenopathy (Fig. 5). The gallbladder was removed together with a portion of liver tissue for diagnostic purposes. Pathology revealed presence of acute-on-chronic cholecystitis, with severe inflammatory changes (lymphocytes, neutrophils, plasma cells, eosinophils), focal ulceration and necrosis, focal adenomyosis. No malignant atypic cells were described (Fig. 6). The patient was discharged after the intervention without complications and he is now on follow-up for the bladder cancer, without recurrence of gastrointestinal symptoms.

## Discussion

Conventionally, a gallbladder wall more than 3 mm by ultrasound is considered thickened. Different pathologic conditions cause the thickening of the gallbladder wall, which are usually classified according to the pattern of the morphological alteration: diffuse and focal thickening (Table 1). Acute cholecystitis is the most frequent inflammatory condition of the gallbladder and is associated in 90–95% of cases with presence of stones (cholelithiasis) [3]. Diagnostic criteria for acute cholecystitis [4] are based on the presence of: (1) local inflammation (Murphy’s sign, RUQ mass, pain, tenderness); (2) systemic inflammation (fever, elevated CRP, elevated WBC); (3) imaging findings (wall thickening > 3 mm, wall edema, gallbladder distension > 40 mm, positive sonographic Murphy sign, pericholecystic and perihepatic fluid) [5]. The differential diagnosis of gallbladder wall thickening includes gallbladder carcinoma, an uncommon malignancy arising from the gallbladder. Advanced age and female gender are predisposing conditions. Gallstones are the strongest risk factor, followed by porcelain gallbladder, gallbladder polyps and primary sclerosing cholangitis [6]. Ultrasound findings of gallbladder carcinoma are: mass replacing the gallbladder with liver infiltration, asymmetric mural thickening, intraluminal polypoid mass. Since the poor prognosis associated to the condition, a diagnostic delay may increase morbidity and mortality. Conversely, an early diagnosis, especially when the tumour is confined to the wall, improves survival. Diagnostic imaging does not distinguish between benign and malignant etiologies in all cases, although presence of certain features provides important clues for diagnosis [2]. Focal wall thickening and polypoid lesions > 10 mm are concerning features and MRI imaging may reveal lymphnode involvement in patients with suspicious ultrasound findings [7]. The use of contrast-enhanced ultrasound (CEUS) in gallbladder pathology is supported by recent guidelines [8]. CEUS features suggestive of malignancy are: washout within 35 s after contrast agent injection, disruption of gallbladder wall and infiltration of the adjacent liver parenchyma [8]. In our patient, the preoperative imaging findings coupled with the clinical picture, atypical for acute cholecystitis, were highly suggestive of a malignant lesion, but the final diagnosis by pathology proved the prediction wrong. Given the diagnostic overlap between benign and malignant gallbladder lesions [9] and the aggressive nature of gallbladder cancer, cholecystectomy is advisable in equivocal cases to solve the diagnostic dilemma.

**Table 1:** Etiology of gallbladder wall thickening (diffuse and focal involvement)

Diffuse wall thickening	Focal wall thickening
Acute cholecystitis (calculous, acalculous)	Polyps (adenomatous, cholestral)
Chronic cholecystitis (xantogranulomatous)	Polyps (adenomatous, cholesterol)
Liver disease (hepatitis, cirrhosis, portal hypertension)	Malignancy (primary gallbladder carcinoma, metastases)
Extra cholecystic inflammation (pancreatitis, colitis, peritonitis, pyelonephritis)	Focal adenomyomatosis
Systemic diseases (congestive heart failure, renal failure, sepsis, hypoalbuminemia)	Focal xantogranulomatous cholecystitis
Malignancy (primary gallbladder carcinoma, lymphoma)	
Adenomyomatosis	
Pseudo thickening (contracted state)	
Atypical infection (tuberculous, dengue hemorragic fever)	
